# Meta-Analysis of the Effect of Nitric Oxide Application on Heavy Metal Stress Tolerance in Plants

**DOI:** 10.3390/plants12071494

**Published:** 2023-03-29

**Authors:** Xiaoxiao Liu, Di Gong, Qingbo Ke, Lina Yin, Shiwen Wang, Tianpeng Gao

**Affiliations:** 1 School of Biological and Environmental Engineering, Academy of Eco Xi’an, Xi’an University, Xi’an 710065, China; 2 Shaanxi Provincial Natural Forest Protection Project Management Center, Xi’an 710082, China; 3 Institute of Soil and Water Conservation, Chinese Academy of Sciences and Ministry of Water Resources, Yangling 712100, China; 4 State Key Laboratory of Soil Erosion and Dryland Farming on the Loess Plateau, Institute of Soil and Water Conservation, Northwest A&F University, Yangling 712100, China; 5 Engineering Center for Pollution Control and Ecological Restoration in Mining of Gansu Province, Lanzhou City University, Lanzhou 730070, China

**Keywords:** heavy metal stress, oxidative stress, nitric oxide, stress alleviation

## Abstract

Substantial single-species studies have reported the facility of nitric oxide (NO) in alleviating heavy metal-induced stress in plants. Understanding the mechanisms of NO-involved stress alleviation is progressing; however, a quantitative description of the alleviative capacity of NO against heavy metal stress is still lacking. We combined the results of 86 studies using meta-analysis to statistically assess the responses of heavy metal-stressed plants to NO supply across several metal stresses and plant families. The results showed that plant biomass was consistently improved following NO supply to metal-stressed plants. NO played an important role in mitigating oxidative damage caused by heavy metal stress by significantly stimulating the activities of antioxidant enzymes. Moreover, NO supply consistently increased the Ca, Fe, and Mg contents in both leaves and roots. Plant tissues accumulated less heavy metals when exposed to heavy metal stress after NO addition. Additionally, the best concentration of SNP (an NO donor) for hydroponic culture is in the range of 75–150 μM. We further confirmed that NO application can generally alleviate plant heavy metal stress and its action pathway. The results presented here can help guide future applications of NO as a plant growth regulator in agriculture and breeding plants for heavy metal stress tolerance.

## 1. Introduction

Nowadays, with the rapid development of industrialization and increasingly frequent anthropogenic activities, heavy metal contamination is causing strongly destructive effects on the balance of ecosystems, disturbing not only the development of agriculture but also food production and security [1]. Plants are sessile organisms that easily suffer from heavy metal stress in their life cycles. Once concentrations exceed their safety thresholds, heavy metals are phytotoxic, disturbing plant physiology and metabolism and limiting plant growth and development [2]. Heavy metals have the characteristics of mobility, bioaccumulation, and bioavailability; thus, they enter into plants easily and pose a serious threat to human health through the food chain [3].

Plants growing under heavy metal stress conditions have the symptoms of necrosis, chlorosis, nutrient deficiency, stunted growth, and reduced biomass likely because heavy metals denature various important enzymes and interfere with the substitution reactions of essential metal ions in plants [4]. Moreover, when plants are exposed to heavy metal stress, reactive oxygen species (ROS) are inevitable produced, leading to serious oxidative damage. Hydrogen peroxide (H_2_O_2_), malondialdehyde (MDA), and proline are common oxidative stress markers, which are all increased after exposure to heavy metal stress [5]. Along with the production of ROS, a process that simultaneously happens in plants, heavy metal stress causes the synthesis of enzymatic and non-enzymatic antioxidants, such as superoxide dismutase (SOD), catalase (CAT), ascorbate peroxidase (APX), peroxidase (POD), ascorbate (AsA), and glutathione (GSH) [6,7]. Additionally, heavy metal stress can interfere with the absorption of Ca, P, K, Mg, and other metals, disturbing the balance of elements in plants [4]. Beyond a doubt, heavy metals have detrimental effects on plant growth; therefore, attempting to reduce heavy metal contamination is urgently needed. The application of an efficient and eco-friendly approach to reduce heavy metal accumulation and toxicity in metal-stressed plants is a useful strategy to solve the problem.

As a crucial gaseous signaling molecule, nitric oxide (NO) plays an important role in multiple tissues of plants, modulating a large number of physiological and biochemical processes [8,9,10]. It has been reported that NO is involved in the regulation of diverse plant responses to many abiotic stresses [11,12]. Recently, an increasing number of studies have indicated the effects of exogenous application of NO on ameliorating the toxicity induced by heavy metals such as cadmium (Cd) [13,14], arsenic (As) [15], copper (Cu) [16,17], lead (Pb) [18], nickel (Ni) [19], and zinc (Zn) [20]. However, due to inadequate molecular information, most of the current NO research in plants bank on exogenous application of NO donors in laboratory experiments, and this approach has been used to mimic an endogenous NO-related response.

Exogenous NO is routinely applied to a variety of grain crops under heavy metal stress, for example, rice, wheat, and maize, to alleviate the negative effects of heavy metal stress in plants [21,22]. According to a large number of previous studies, several mechanisms are involved in the mitigation of heavy metal stress by NO: (a) acceleration of stress responses by indirectly increasing antioxidant production. Heavy metal stress generally causes vast ROS formation [23] and a large amount of evidence has reported that heavy metal toxicity is partially due to oxidative damage [24]. NO participates in ROS metabolism in plants [25]. It is widely accepted that exogenous application of NO protects plants against oxidative damage by increasing the antioxidant content as well as the activities of antioxidative enzymes [26,27,28,29]; (b) regulation of the uptake and accumulation of toxic or other elements, thus changing the element distribution and storage location in plants. It has been reported that exogenous NO increased Cd accumulation in the root cell wall while decreasing Cd accumulation in rice leaves [30]. In the roots of *Medicago truncatula*, NO mitigated Cd stress by decreasing the Cd content and enhancing the absorption of K^+^ and Ca^2+^ [31]. The possible anti-toxicity functions of NO include recovery of crown root number, plant active excretion, metal binding, co-precipitation and chelation, compartmentalization in vacuoles, etc. [32]; (c) effects on plant signaling cascades, thus regulating the expression of heavy metal stress-related genes in plants, increasing the Ca^2+^ content, and stimulating P5CS and ferritin gene expression [9,33,34].

Plants have evolved several strategies to alleviate metal-induced stress, including the strategy employed in some hyperaccumulator species, such as Indian mustard (*Brassica juncea* L.) of the family Brassicaceae, finger millet (*Eleusine coracana*) of the family Poaceae, sunflower (*Heliannthus annuus* L.) of the family Asteraceae, and rattlebush (*Sesbania drumondiiallus*) of the family Fabaceae. These are identified as species showing high tolerance to heavy metal stress and are, therefore, able to accumulate more metals, such as Cd, Cu, Ni, Zn, and Pb, to a certain extent [35,36,37,38]. Plant hypertolerance to heavy metals has the characteristics of hyperaccumulation of metals and the presence of a strong antioxidant defense system and heavy metal detoxification system [36,39,40]. Plant tolerance to heavy metals varies from species to species [4]. Therefore, the heavy metal hyperaccumulation capacity and plant family could be factors affecting the effect of NO on alleviating heavy metal stress in plants.

Diverse stress types could have different responses in how NO alleviates heavy metal-stressed plants. NO participates in diverse mechanisms of stress mitigation according to the type of stress [12]. An example is the antioxidant activity of SOD, which is significantly decreased or increased when different heavy metal-stressed plants are applied with NO [41,42,43,44]. Another factor that possibly affects the mitigation of heavy metal stress by NO is the plant part (shoot or root). Different responses have been noted between leaves and roots when NO is applied to heavy metal-stressed plants [45]. It has been reported that NO application to Cd-stressed *Typha angustifolia* caused increased Cd contents in the roots compared with the shoots [46]. Knowledge about plant part responses may provide a further understanding of the mechanisms underlying the alleviative role of NO against a range of heavy metal stresses.

The various single-species studies give the opportunity to determine the consistent function of NO across a diverse range of heavy metal stress types and species. However, there are still a lot of uncertainties due to differences between diverse studies, such as the concentrations of sodium nitroprusside (SNP, a widely used NO donor that provides a persistent pattern of NO release compared with other donors) that were used, covering from 0.1 μM to 1 mM [19,47]. A numerical assessment is still lacking and the results of exogenous application of NO in heavy metal stress alleviation between different heavy metal stress types and plant families are still largely unknown. Meta-analyses can conduct quantitative comparisons of multiple data extracted from numerous studies [48]. Through quantitative analysis, we sought to focus on and definitively determine whether plant biomass and chlorophyll content were increased after exogenous application of NO to heavy metal-stressed plants and whether this consistently occurred through the alleviation of oxidative stress as well as the accumulation of nutrient elements and heavy metal elements. Additionally, the different responses were analyzed among different stress types. Considering all heavy metal stress types that could possibly cause oxidative damage, we predicted that heavy metal stress type would not be a significant explanatory factor. We also analyzed whether plant family is a crucial explanatory factor because some plant species are hyperaccumulators. Subsequently, we explicitly analyzed the optimum concentration range of SNP applied to plants in laboratory experiments. Thus, the objective of this study was to investigate the responses of heavy metal-stressed plants to NO supply across several metal stresses and plant families, which could contribute to a better understanding of the basic mechanisms of NO in heavy metal stress adaptation in plants.

## 2. Results

### 2.1. Dataset

The dataset used across the meta-analysis included 6 heavy metal stresses, 42 plant species, and 5 plant families (Supplementary Data, summary of plant species and results of different analysis, e.g., plant growth, oxidation levels, element content, etc.) (Table 1). The majority of species were agricultural plants. A large number of experiments were carried out by hydroponic culture (64 studies, accounting for 74%), while a small part used seedling spraying, irrigation, or seed soaking. If the studies were performed in the same plant family or the same stress, differences in growing media, study duration, and stress intensity were not considered. The metal stress exposure most frequently used in all studies was Cd stress (n = 36 papers), while others were As stress (n = 21), Cu stress (n = 12), Ni stress (n = 4), Pb stress (n = 7), and Zn stress (n = 7). Considering plant families, Poaceae was best represented with 35 studies (7 species), followed by Fabaceae in 16 studies (10 species), Solanaceae in 9 studies (4 species), Asteraceae in 7 papers (4 species), and Brassicaceae in 8 papers (5 species).

### 2.2. Plant Growth and Chlorophyll Concentration

**Figure 1:**
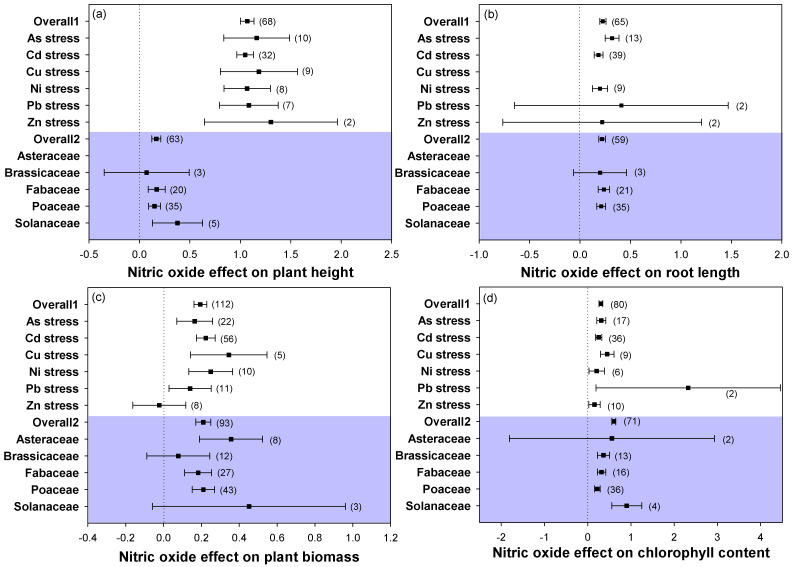
Plots of effect sizes for the effect of NO on (**a**) plant height, (**b**) root length, (**c**) plant biomass, and (**d**) chlorophyll content. Overall1 and overall2 indicate the mean (summary) effect size of all heavy metal stresses and plant families, respectively. The numbers in brackets specify the number of data points. The black square block with the error bar indicates the mean response size with a 95% confidence interval, and the dashed vertical line shows zero effect.

Overall, combining studies across 6 heavy metal stress types and 5 plant families, application of NO to heavy metal-stressed plants dramatically increased plant height, root length, and biomass (Figure 1a–c). However, not all groups showed a prominent increase in plant height, root length, and plant biomass with NO supply. Brassicaceae showed no significant differences as compared with other plant families. No significant increase in root length was found under Zn and Pb stresses after NO application nor in plant biomass under Zn stress for the families Brassicaceae and Solanaceae.

Analyses showed that the addition of NO to heavy metal-stressed plants dramatically increased the chlorophyll concentration (Figure 1d). All of the heavy metal stress types and plant families showed a positive response to NO addition in their chlorophyll contents. These results indicated that plant growth and chlorophyll content were promoted with the application of NO under heavy metal stress, although some conspicuous responses of stress type and plant family existed.

### 2.3. Oxidative Stress Markers

**Figure 2:**
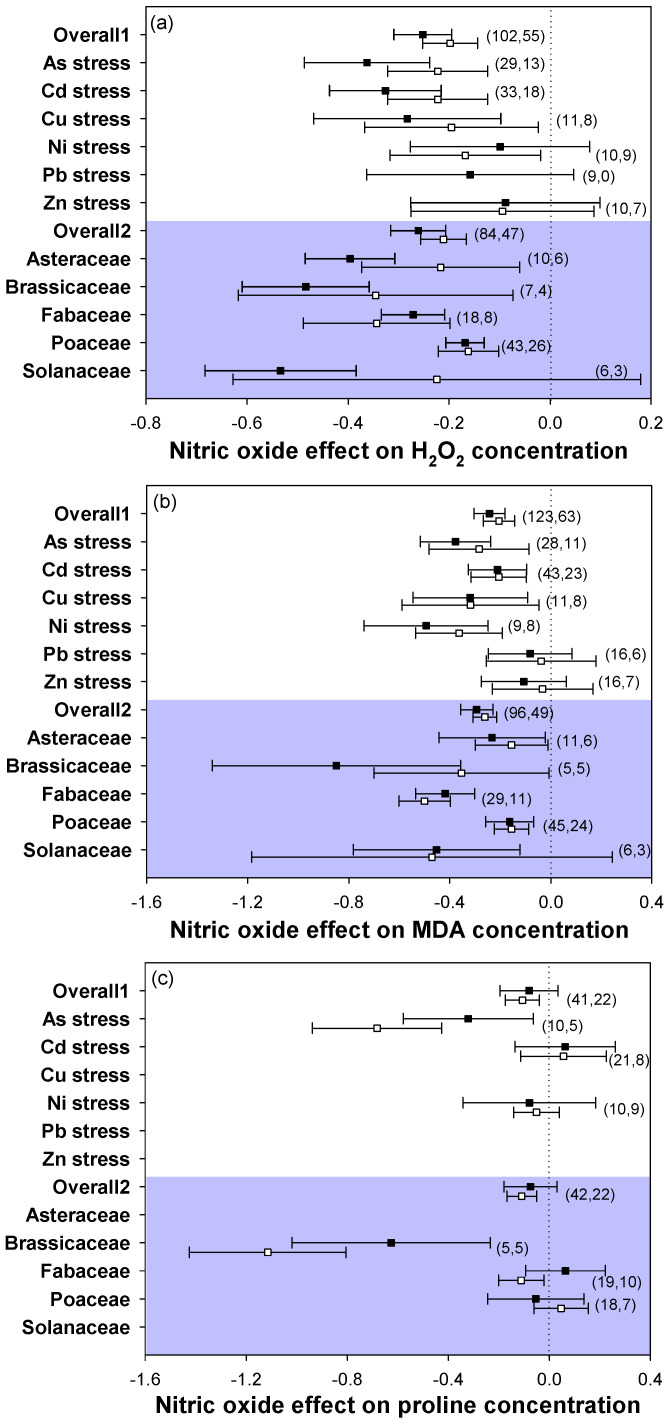
Plots of effect sizes for the effect of NO on oxidative stress markers in heavy metal-stressed plants, including (**a**) H_2_O_2_ concentration, (**b**) MDA concentration, and (**c**) proline concentration. Overall1 and overall2 indicate the mean (summary) effect size of all metal stresses and plant families, respectively. Black and white dots indicate the mean effect sizes for factor groups of leaf and root responses, respectively. The numbers in brackets specify the number of data points. The black square block with the error bar indicates the mean response size with a 95% confidence interval, and the dashed vertical line shows zero effect.

NO application to heavy metal-stressed plants significantly mitigated oxidative damage in both leaves and roots, as shown by the represented oxidative stress markers, such as H_2_O_2_, MDA, and proline (Figure 2a–c). The H_2_O_2_ concentration was significantly reduced by NO application under 3 types of heavy metal stress in both leaves and roots, including As, Cd, and Cu stresses, in 4 plant families, covering Asteraceae, Brassicaceae, Fabaceae, and Poaceae. The MDA concentration showed a consistent trend with H_2_O_2_, which significantly decreased following NO addition in most heavy metal stress types and plant families, but not in Pb and Zn stresses and the family Solanaceae. The proline content varied a lot among stress types and plant families, showing negative, positive, or no effects of NO supply on metal-stressed plants between stress types and families. Although several of the effect sizes of MDA in both leaves and roots were positive, they were not significant. Overall, NO consistently mitigated oxidative stress, with heavy metal stress type and plant family considered as explanatory factors.

### 2.4. Antioxidant Responses

**Figure 3:**
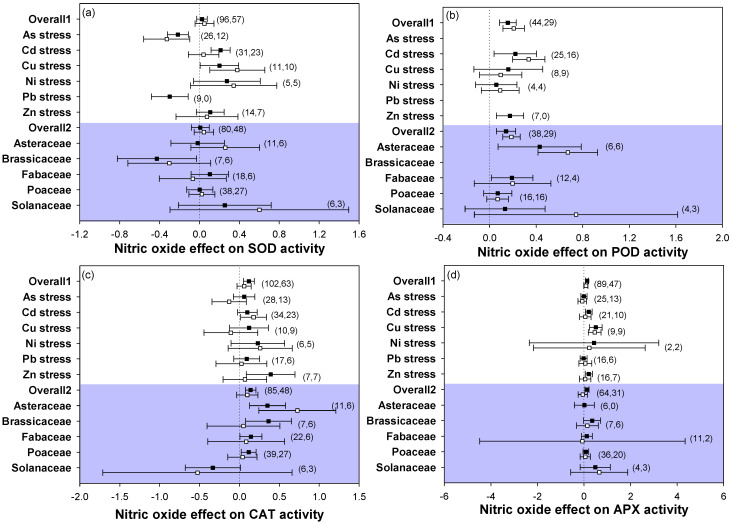
Plots of effect sizes for the effect of NO on enzymatic antioxidant activities of heavy metal-stressed plants, including (**a**) SOD activity, (**b**) POD activity, (**c**) CAT activity, and (**d**) APX activity. Overall1 and overall2 indicate the mean (summary) effect size of all metal stresses and plant families, respectively. Black and white dots indicate the mean effect sizes for factor groups of leaf and root responses, respectively. The numbers in brackets specify the number of data points. The black square block with the error bar indicates the mean response size with a 95% confidence interval, and the dashed vertical line shows zero effect.

For enzymatic antioxidant responses, the addition of NO to heavy metal-stressed plants increased the activities of SOD, POD, CAT, and APX in the leaves or roots in most stress types and plant families (Figure 3a–d). Meanwhile, we also found that NO addition significantly reduced SOD activity in both leaves and roots under As stress, in leaves under Pb stress (no data for SOD activity were found for roots under Pb stress), as well as in plants of the family Brassicaceae. Additionally, for CAT responses in As- or Cu-stressed plants, supplying NO caused different results in leaves and roots, with decreased CAT activity in roots while it was increased in leaves. In the family Solanaceae, CAT activity was also reduced in both leaves and roots.

**Figure 4:**
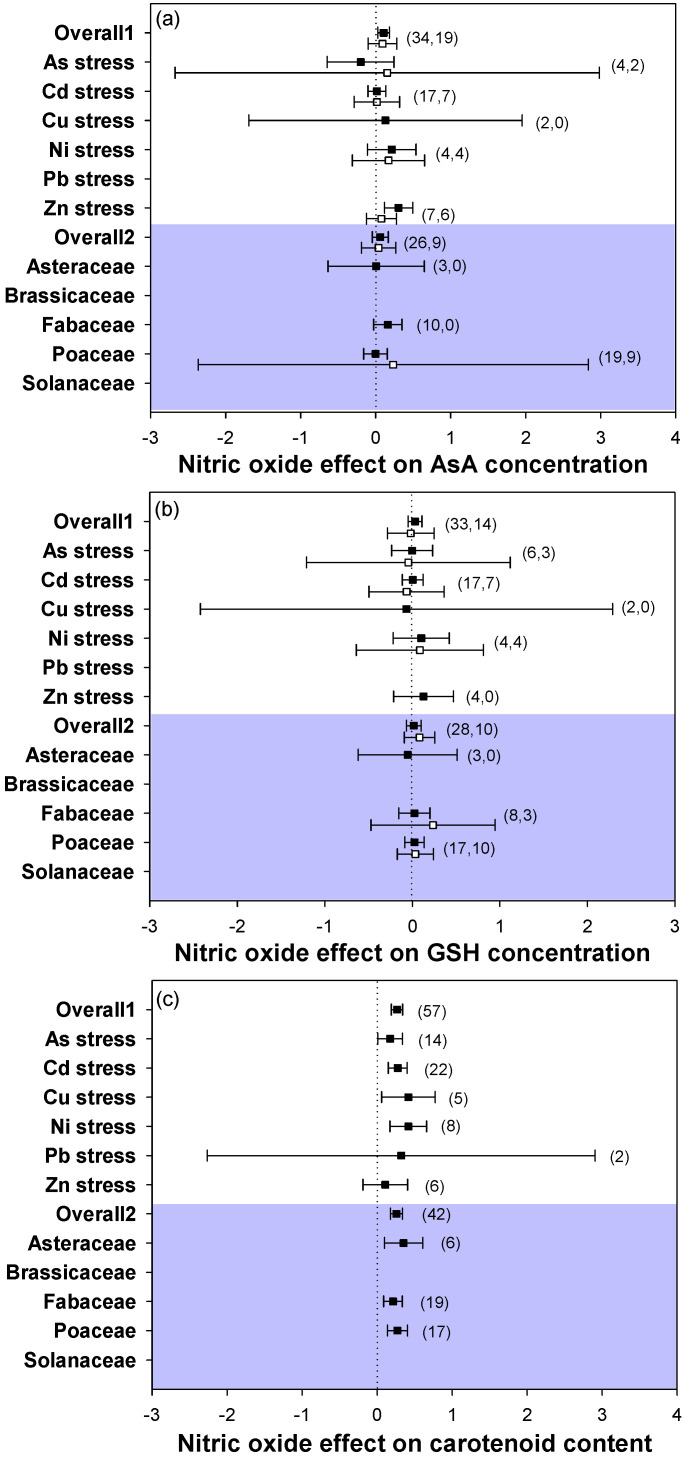
Plots of effect sizes for the effect of NO on antioxidant contents of heavy metal-stressed plants, including (**a**) AsA concentration, (**b**) GSH concentration, and (**c**) carotenoid content. Overall1 and overall2 indicate the mean (summary) effect size of all metal stresses and plant families, respectively. Black and white dots indicate the mean effect sizes for factor groups of leaf and root responses, respectively. The numbers in brackets specify the number of data points. The black square block with the error bar indicates the mean response size with a 95% confidence interval, and the dashed vertical line shows zero effect.

For non-enzymatic antioxidant responses, NO supply increased the contents of antioxidant substances, as shown by increases in the contents of AsA, GSH, and carotenoids under heavy metal stress conditions (Figure 4a–c). However, the response of AsA concentration in leaves under As stress was negative. Moreover, including all the analyzed heavy metal stress types and plant families in both leaves and roots, the changes in AsA and GSH contents showed consistent trends but did not have significant effects. In addition, NO application dramatically increased the carotenoid contents across heavy metal stress types and plant families. Therefore, these results showed that there were consistent responses in antioxidant ability with NO application under heavy metal stress conditions.

### 2.5. Element Content

**Figure 5:**
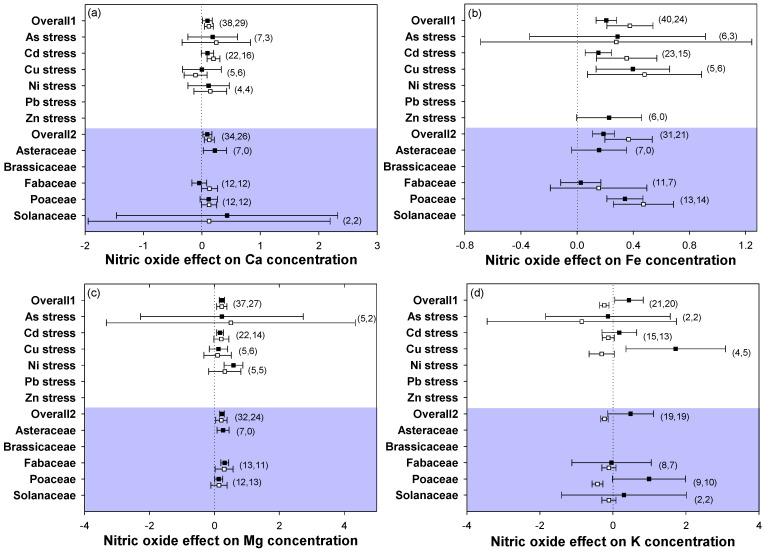
Plots of effect sizes for the effect of NO on element accumulation of metal-stressed plants, including (**a**) Ca, (**b**) Fe, (**c**) Mg, and (**d**) K. Overall1 and overall2 indicate the mean (summary) effect size of all metal stresses and plant families, respectively. Black and white dots indicate the mean effect sizes for factor groups of leaf and root responses, respectively. The numbers in brackets specify the number of data points. The black square block with the error bar indicates the mean response size with a 95% confidence interval, and the dashed vertical line shows zero effect.

The overall effect sizes showed that NO supply consistently increased the Ca, Fe, and Mg contents in both leaves and roots, while it decreased the K content in roots in all heavy metal stress types and plant families (Figure 5a–d). Similarly, the K content in leaves was increased after the addition of NO under heavy metal stress. For Ca, Mg, and K contents, the effect sizes showed no significant responses. Supplying NO to stressed plants significantly increased the Fe contents both in leaves and roots. These results indicated that plants could consistently regulate the distribution of some elements when plants were supplied with NO under heavy metal stress conditions. Additionally, the changes in the element contents in leaves and roots were maintained consistently, except for the K element concentration.

### 2.6. Heavy Metal Accumulation

**Figure 6:**
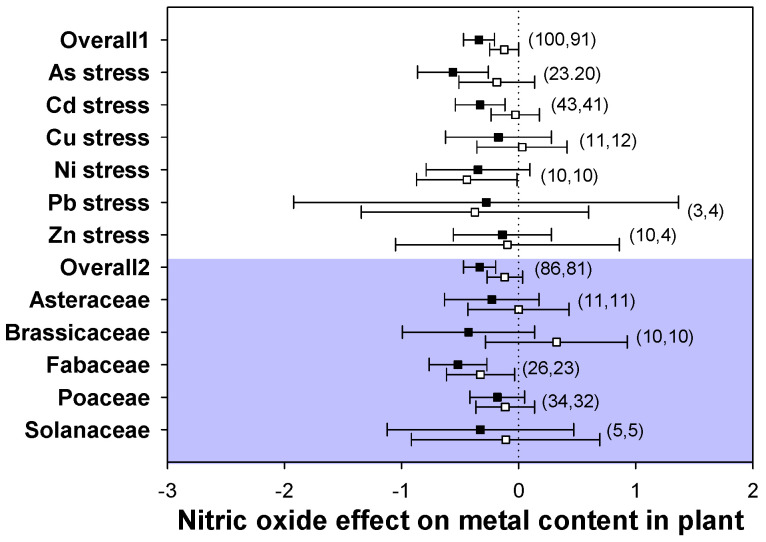
Plots of effect sizes for the effect of NO on metal content in plants. Overall1 and overall2 indicate the mean (summary) effect size of all metal stresses and plant families, respectively. Black and white dots indicate the mean effect sizes for factor groups of leaf and root responses, respectively. The numbers in brackets specify the number of data points. The black square block with the error bar indicates the mean response size with a 95% confidence interval, and the dashed vertical line shows zero effect.

The all effect sizes showed that NO application consistently decreased the metal contents across the tested heavy metal stress types and plant families in both leaves and roots, although the accumulation of metal ions in roots was not significantly affected (Figure 6). The metal content in leaves varied a lot among different types of heavy metal stresses and plant families, especially in As and Cd stresses and the family Fabaceae. Meanwhile, for the metal content in roots, there were no dramatic moderators to explain the heterogeneity. The significant reduction in metal content was maintained in Fabaceae, while the other 4 plant families did not show marked effects in both leaves and roots after the addition of NO. These results suggested that NO has potential value for decreasing metal accumulation in plants under heavy metal stress, but it only shows a significant effect in leaves and not in roots.

### 2.7. The Best Concentration of SNP for Hydroponic Experiments

**Figure 7:**
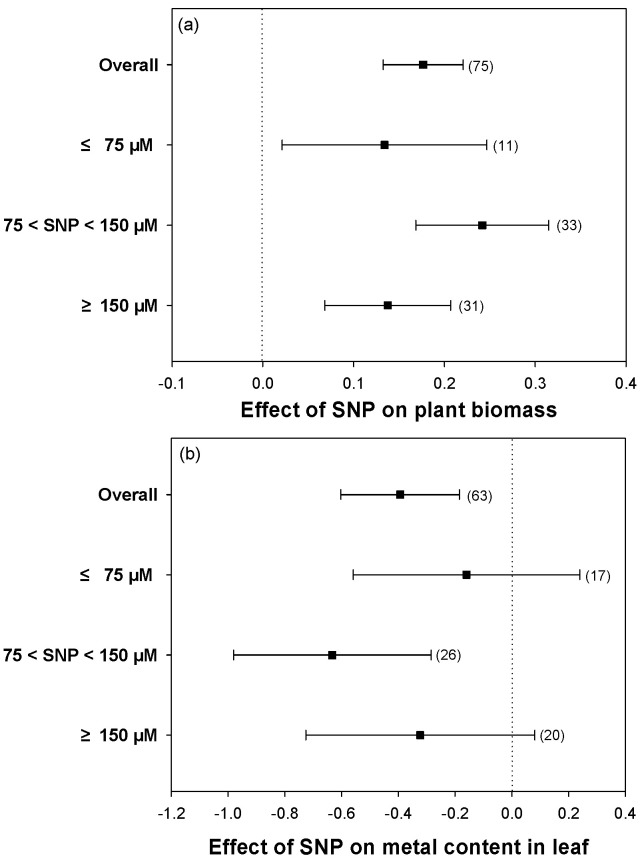
Plots of effect sizes for the effect of different SNP concentrations on (**a**) plant biomass and (**b**) metal content in leaves. The numbers in brackets specify the number of data points. The black square block with the error bar indicates the mean response size with a 95% confidence interval, and the dashed vertical line shows zero effect.

Due to most of the studies being conducted at the laboratory level using hydroponic culture, the responses at different SNP concentrations were also analyzed. Based on the data collected, the SNP concentrations were divided into three categories, including ≤75 μM, 75 < SNP < 150 μM, and ≥150 μM. Different concentrations of SNP added to heavy metal-stressed plants improved the biomass of plants (Figure 7a). Compared with other SNP concentrations, concentrations of SNP between 75 and 150 μM alleviated heavy metal stress more effectively. Meanwhile, the heavy metal content in leaves was significantly reduced at the same SNP concentrations (75 < SNP < 150 μM), leading to higher plant biomass (Figure 7a,b). On the other hand, too high concentrations of SNP possibly had a less mitigative role against heavy metal stress.

## 3. Discussion

In the present study, NO application contributed to higher plant biomass in diverse heavy metal stress types and plant families, indicating that the alleviative role of NO against heavy metal stress is a definite fact. Such an obvious effect of NO on alleviating heavy metal stress has a close relationship with its effect on reducing heavy metal stress-induced oxidative damage, which was found across all tested stress types and plant families in our study, since the antioxidant abilities were significantly improved following NO application (Figure 2, Figure 3 and Figure 4). Additionally, the alteration of other nutrient and heavy metal contents could also contribute to the beneficial effect of NO against heavy metal stress (Figure 5 and Figure 6).

### 3.1. Application of NO Improved Plant Growth against Heavy Metal Stress

It has been suggested that NO has the ability to counteract the adverse effects of heavy metal stress on plant growth and development [27,49]. In the current meta-analysis, the results showed that the biomass and plant growth were increased across diverse heavy metal stresses and plant families following NO supply (Figure 1a–c). The NO-mediated increase in the biomass of heavy metal-stressed plants can be partially ascribed to the increased chlorophyll contents (Figure 1d). Exogenous addition of NO contributed to enhancing the growth and biomass yield and protected chlorophyll pigments in tomato under Cd stress [50]. However, until now, the NO-involved alleviative mechanisms were still not very clear. Moreover, whether NO participates in these growth and development processes directly as a signaling molecule is still debated and needs further investigation.

### 3.2. Application of NO Reduced Oxidative Damage under Heavy Metal Stress

Although plant response to heavy metal-induced stress could be metal- or species-specific, heavy metal-induced oxidative damage seems to be a common characteristic, with ROS accumulating rapidly under heavy metal exposure as well as in a large amount within a certain time [51,52]. In the current study, the application of NO consistently decreased the oxidative damage, as shown by the decreased H_2_O_2_ and MDA levels in both the leaves and roots of plants subjected to NO across diverse stress types and plant families, indicating that the advantages of NO for the amelioration of heavy metal stress-induced oxidative stress were nearly consistent across stress types and plant families (Figure 2a,b). Proline, as an amino acid, was observed to be accumulated in plants exposed to heavy metal stress [4]. In this study, there were positive response sizes of the NO effect on the proline concentration under Cd stress in the roots of the family Fabaceae and in the leaves of the family Poaceae (Figure 2c). Other groups showed negative response sizes, indicating that NO had no significant effect on plant growth alleviation through decreasing the proline content to alleviate oxidative injury under Cd stress. The positive effect in the roots of Fabaceae and the leaves of Poaceae may be explained by the presence of some heavy metal-tolerant species in these families, such as *Zea mays* of Poaceae [35]. They are metal-tolerant plants that can accumulate high levels of proline [4]. Under As and Ni stresses, the response sizes of proline were negative, indicating that NO could decrease the proline content to diminish oxidative damage under those stresses. These results suggest that exogenous NO-induced alleviation against heavy metal stress could be ascribed to decreasing the concentrations of H_2_O_2_ and MDA in a vast majority of plants but not the proline content. The levels of H_2_O_2_ and MDA can better reflect the alleviating effect of NO against heavy metal stress.

### 3.3. Application of NO Increased Plant Antioxidant Ability under Heavy Metal Stress

A large amount of evidence has proven that NO-mediated protection against heavy metal stress is closely related to improved antioxidant ability [23]. In the present study, the activities of antioxidant enzymes (SOD, POD, and CAT) were increased among stress types and plant families by NO application (Figure 3a–c). The significant increase in SOD, POD, and CAT activities may work as a physical mechanism responsible for the alleviation of heavy metal stress by NO application. Negative response sizes of the activity of SOD under As and Pb stresses were also detected after NO addition, which may be plant species- or stress type-specific. An example was observed in rice, where the SOD activity was prominently decreased in both leaves and roots after application of NO under As stress, while it was increased under Cd stress [43,53]. Under the same heavy metal stress of Pb, the SOD activity in leaves decreased in *Arabidopsis thaliana* (Brassicaceae) but increased in cowpea (Febaceae) with application of NO [44,54]. Similarly, NO application in As-stressed plants caused different responses in the antioxidant system, which could be ascribed to plant species and tested plant organs [23]. Exogenous supply of NO caused a negative effect size of SOD activity in Brassicaceae under heavy metal stress, suggesting that the alleviative effect of NO on heavy metal-induced stress was not dependent on antioxidant ability in Brassicaceae. Additionally, some plant species are hyperaccumulators, such as *Brassica juncea* (Brassicaceae), which show high tolerance to Cd, Cu, Pb, Ni, and Zn stresses (https://en.wikipedia.org/wiki/List_of_hyperaccumulators, accessed on 23 December 2022). This may cause the decrease in SOD activity after NO addition when plants are exposed to heavy metal stress. Importantly, the consistent increase in the activities of POD and CAT among plant families and heavy metal stress types found in this analysis showed that they were the main parameters to evaluate the alleviative role of NO on heavy metal stress. Regarding APX, NO application had a little effect among plant families and stress types (Figure 3d), suggesting that changes in APX cannot explain the role of NO in alleviating heavy metal stress. These results suggested that application of NO significantly alleviated heavy metal stress mainly through increasing the activities of SOD, POD, and CAT.

Regarding the non-enzymatic antioxidant defense system, AsA, GSH, and carotenoids play an important role in sequestering metals and are beneficial for ROS defense [55,56]. In the present study, the results of the meta-analysis showed that the application of NO consistently increased the content of non-enzymatic antioxidants (including AsA, GSH, and carotenoids) under heavy metal stress in all stress types and plant families, but the changes in AsA and GSH were not significantly (Figure 4a–c). This may depend on the responses of the enzymatic antioxidants. It has been reported that an increased content of AsA was observed under Cd stress when plants were treated with NO, which was related to the increased activity of APX [50]. Carotenoids are used to determine the status of the response of non-enzymatic antioxidants, which play an important role in alleviating heavy metal stress [57]. In the current study, the significant effect of NO on non-enzymatic antioxidants was shown in carotenoid content, which had positive response sizes across stress types and plant families (Figure 4c), suggesting that carotenoids may play an important role in the alleviation of heavy metal stress by NO. However, compared with the enzymatic antioxidant responses, the non-enzymatic antioxidant responses were not changed significantly overall when NO ameliorated heavy metal -stressed plants based on the present studies. Taken together, the meta-analysis quantitatively showed the effective role of NO in the alleviation of heavy metal stress across stress types and plant families, especially through the stimulation of the enzymatic antioxidant system.

### 3.4. Application of NO Increased the Contents of Essential Elements While Decreasing the Contents of Heavy Metal Elements under Heavy Metal Stress

Heavy metals have interactions with other elements, and the application of NO may affect the absorption or transportation of other elements. It has been reported that the Cd-caused decrease in the Ca content in roots was reversed by the application of NO in *Trifolium repens* L. [58]. NO participated in the activation or repression of Ca^2+^ influxes under heavy metal-induced stress [59,60]. In the current study, the results showed that the contents of elements Ca, Fe, and Mg showed no marked differences between leaves and roots, which all presented positive effect sizes after NO application, indicating that exogenous application of NO increased the contents of elements Ca, Fe, and Mg in plants (Figure 5a–c). It had been reported that exogenous addition of NO could mitigate Fe absorption, thus the NO mitigation of repressed growth may be related to the increased absorption of macro- and micronutrients [27]. Regarding the K content, it was increased in leaves but reduced in roots with NO addition to heavy metal-stressed plants (Figure 5d). In plants, the movement of K shuttles within plant cells and its uptake from the environment is mediated by K transporters, thus there may be diverse transport rates of the K element between the roots and leaves in plants [61] or K uptake by the roots is controlled by leaf requirement, since it is possible that most of the K uptake by roots is transported to the leaves, which in turn causes a decreased K content in the roots [62]. Moreover, the addition of NO considerably improved the leaf and root K content under Cd and Pb stresses [63], whereas the concentrations of K in both leaves and roots were all decreased by NO application in As-stressed plants [62]. Based on these inconsistent results, the effect of NO application on K content under heavy metal stress still needs further investigation.

In the present study, exogenous application of NO consistently decreased the heavy metal content in both leaves and roots in diverse stress types and plant families in laboratory experiments (Figure 6). Compared with the heavy metal content in leaves, the content of heavy metals in roots was less reduced, with no significant changes. NO did not induce a marked decrease in the heavy metal content in stressed plants. However, plants may possibly reduce the transport of heavy metals from roots to leaves by NO addition, therefore diminishing the levels of metals in the leaves. Plant roots are the part directly in contact with heavy metals, so root uptake of metals is important for the metal content in plant tissues [30]. NO, as a signaling molecule, may possibly be involved in the translocation of metals from roots to leaves and be restricted by internal barriers defending the leaves [11]. It has also been reported that exogenous NO inhibited the root-to-leaf translocation of Cd, thus resulting in lower Cd accumulation in leaves [64]. Therefore, NO application may participate in decreasing the content of heavy metals in plant tissues, which enhances the tolerance of plants against heavy metal stress.

### 3.5. Application of NO Is a Useful Approach in Alleviating Heavy Metal Stress

In this study, we collected the majority of agricultural species, which represented different taxonomic groups, covering Asteraceae, Brassicaceae, Fabaceae, Poaceae, and Solanaceae (Table 1). Consistent responses demonstrated that the alleviative functions of exogenous NO against heavy metal stress were universal among these plant species. Based on various single studies, this meta-analysis provided strong evidence that in laboratory research, NO could be an important molecule for metal stress alleviation in a large number of plant species. In addition, the most used concentration of an NO donor (SNP) was in the range of 75–150 μM in hydroponic culture [65,66,67]. Meanwhile, a high concentration of SNP, such as 400 μM, had no mitigative effect on plant growth under heavy metal stress [68]. In the analyzed studies, there were several approaches taken to apply SNP to plants, including adding SNP to solution in hydroponic experiments, irrigating soil with SNP solution, spraying SNP solution on plant leaves, and seed soaking; these applied approaches are limited to laboratory studies. Among those approaches, the most widely used method was the application of SNP in hydroponic culture, with all of these methods inducing the alleviative effect on plant growth against heavy metal stress. Similar to previous studies, our meta-analysis and regression analysis results also showed that an SNP concentration of 75–150 μM had the best alleviative effects on heavy metal stress in hydroponic experiments (Figure 7a,b). The availability of more data on SNP spraying of seedlings or seed soaking studies in field research would provide a better understanding of the function of NO in mitigating heavy metal stress in agricultural crops.

## 4. Materials and Methods

### 4.1. Data Collection

To collect research papers in which heavy metal-stressed plants were grown with and without NO application, a literature search was carried out in the Web of Science citation database (ISI, Philadelphia, PA, USA) using ((alleviat* or mitigate* or amend* or ameliorat* or stimulat* or contribut* or attenuat* or protect*) not disease not infection not insect not herbiv* not fung* not human not animal*) AND (heavy metal* or heavy-metal or metals or cadmium or ‘Cd’ or arsenic or ‘As’ or copper or ‘Cu’ or lead or ‘Pb’ or nickel or ‘Ni’ or zinc or ‘Zn’) in the topic AND (nitric oxide or ‘NO’ or nitric oxide donor or sodium nitroprusside or ‘SNP’) in the topic, the * represents extensible letters. The studies were further selected according to the domains of the journals, such as plant science, environmental science, ecology, soil science, horticultural, forestry, etc., and the contents of the abstract. In addition, to avoid publication bias, the following criteria were used to select studies for further analysis: (a) at least one single type of heavy metal stress or multiple heavy metal stresses but no interactions between different stresses; (b) the experimental design should include two potential treatments (−NO + stress, +NO + stress); (c) the data in the study should include the mean, replicate size, and standard deviation (or standard error) for the treatments. In total, information from 86 papers was collected for analysis, which covered the stresses of As, Cd, Cu, Ni, Pb, and Zn.

### 4.2. Data Analysis

The data were either accessible from tables or extracted from figures using Web Plot Digitizer [69]. Some data of treatments were excluded because of their small sample sizes that could not satisfy meta-analysis. All the subsets of data were listed and the parameters measured most frequently were selected. Parameters were used in the analyses included plant height, root length, plant biomass, chlorophyll content, and carotenoid content, and both shoot and root responses were used in the analyses of oxidative stress markers (including H_2_O_2_, MDA, and proline content), enzymatic antioxidant activities (including SOD, POD, CAT, and APX), non-enzymatic antioxidant contents (including AsA, GSH), heavy metal element content, and other element content (including Ca, Fe, Mg, and K). Considering that some plant families are hyperaccumulators and possibly have significant differences in their response to heavy metal stress, we classified the plant cultivars into five plant families (Asteraceae, Brassicaceae, Fabaceae, Poaceae, and Solanaceae). Single plant cultivars were excluded from the analysis. We tested the responses of heavy metal stress type and plant family as moderators, for which the most data had been collected, although some heavy metal stress and family data could not be tested in the same way as there was not enough data.

Meta-analysis was conducted using the Meta Win 2.0 statistical program. Based on a random effects model in order to make different kinds of experiments comparable, the response ratio (R) of each variable in the individual studies was calculated from the pair of treatments (−NO + Stress (Xc), +NO + Stress (Xe)) to represent the size of the effect [70], using Equation (1):

lnR = ln(Xe/Xc) = ln(Xe) − ln(Xc)(1)

Variance estimations for each study were represented as V, using the following Equation (2):

V = SDc^2^/(Nc × Xc^2^) + SDe^2^/(Ne × Xe^2^) (2)

Xe, Xc, SDe, SDc, Ne, and Nc are independent mean values, standard deviations, and sample sizes in the experimental and control groups, respectively.

Under the assumption that differences among studies within a class were due to both sampling error and random variation, we used the random effects model to analyze the effect value and variance. A positive effect value indicated that the response measured in treatment was higher than that of the control, while a negative value indicated the opposite. The 95% confidence intervals around the effect size were used to determine whether effect sizes for each factor (heavy metal stress type and plant family) were significantly different from zero; if the 95% confidence intervals did not overlap with zero, the responses were significant.

## 5. Conclusions

Heavy metals cause a large area of contamination, which is a fast-growing environmental issue that poses a fatal threat to ecological systems and humans worldwide. NO, as a gas molecule, should be paid more attention for its alleviative role in heavy metal-stressed plants by exogenous addition of NO donors in such an increasingly contaminated environment. The current meta-analysis provides an integrative and quantitative study that shows whether and how NO plays an immeasurable role in plant heavy metal stress mitigation in laboratory experiments. It showed that NO application significantly alleviated oxidative damage caused by heavy metal stress by stimulating the activation of many enzymatic and non-enzymatic antioxidants in plants, especially the enzymatic antioxidant system, which consistently improved biomass and plant growth. NO supply also consistently increased the contents of nutrient elements Ca, Fe, and Mg in both leaves and roots, decreased the K content in roots, and diminished concentrations of heavy metal elements in plants. The effective concentration of SNP against heavy metal stress is in the range of 75–150 μM in hydroponic culture. Most importantly, the current research is limited to laboratory experiments, and there may still be a certain gap between practical application in agriculture and laboratory results. In the future, we should pay more attention to verifying whether SNP or other NO donors can be widely used in the practice of agriculture, so as to provide an effective approach to alleviating heavy metal toxicity in crop production.

## Figures and Tables

**Table 1:** Species and plant families included in the meta-analysis.

Family	Species
Asteraceae	*Carthamus tinctorius* (safflower), *Helianthus annuus* (sunflower), *Lactuca sativa* (lettuce), *Matricaria chamomilla* (chamomile)
Brassicaceae	*Arabidopsis thaliana*, *Brassica juncea* (mustard), *Brassica napus* (canola), *Nasturtium officinale* (watercress), *Isatis cappadocica*
Fabaceae	*Arachis hypogaea* (peanut), *Lupinus termis* (lupine), *Medicago sativa* (alfalfa), *Medicago truncatula* (alfalfa), *Phaseolus vulgaris* (bean), *Pisum sativum* (pea), *Trifolium repens* (white clover), *Vicia faba* (faba bean), *Vigna radiata* (mungbean), *Vigna unguiculata* (cowpea)
Poaceae	*Eleusine coracana* (finger millet), *Festuca arundinacea* (tall fescue), *Hordeum vulgare* (hulless barley), *Lolium perenne* (ryegrass), *Oryza sativa* (rice), *Triticum aestivum* (wheat), *Zea mays* (maize)
Solanaceae	*Capsicum annuum* (pepper), *Lycopersicon esculentum* (tomato), *Nicotiana tabacum* (tobacco), *Solanum lycopersicum* (tomato)

## Data Availability

Not applicable.

## Supplementary Materials

The following supporting information can be downloaded at: https://www.mdpi.com/article/10.3390/plants12071494/s1. Table S1: The dataset used across the meta-analysis included 6 heavy metal stresses, 42 plant species, and 5 plant families.

## Abbreviations

As	arsenic
Cd	cadmium
Cu	copper
Ni	nickel
Pb	lead
Zn	zinc
NO	nitric oxide
SNP	sodium nitroprusside
SOD	superoxide dismutase
CAT	catalase
APX	ascorbate peroxidase
POD	peroxidase
AsA	ascorbate
GSH	glutathione
H_2_O_2_	hydrogen peroxide
MDA	malondialdehyde
